# Effect of inhalation application of lavender essential oil on slaughter and carcass characteristics and serum biochemistry in Texas quail

**DOI:** 10.1016/j.psj.2025.104828

**Published:** 2025-03-29

**Authors:** Emre ARSLAN, Irem AYRAN COLAK, Rahile OZTURK, Irem BAYAR, Sadiye Ayse CELİK, Tuba BAYIR

**Affiliations:** a Selçuk University, Faculty of Veterinary Medicine, Department of Animal Science, Konya, Türkiye; b Selçuk University, Faculty of Agriculture, Department of Field Crops, Konya, Türkiye; c Selçuk University, Faculty of Science, Department of Biology, Konya, Türkiye; d Selçuk University, Faculty of Veterinary Medicine, Department of Biochemistry, Konya, Türkiye; e Fırat University, Faculty of Veterinary Medicine, Department of Biometrics, Elazığ, Türkiye

**Keywords:** Lavender, Inhalation, Texas quail, Carcass, Metabolism

## Abstract

The aim of this study is to determine the effect of the lavender essential oil inhalation on slaughter and carcass characteristics and biochemical parameters of Texas quails. The material of the study consisted of 72 Texas quails aged between 28 and 42 days and the application was carried out in a 2×2 trial set-up. Inhalation application was carried out in the 4×4 m poultry breeding rooms in the Alternative Poultry Unit at the Prof. Dr. Hümeyra Özgen Research and Application Farm. Lavender essential oil, extracted by distillation, was applied to Texas quails at solution of 0.1 % concentration for 1 h per day for 14 days. A total of 12 quails, including 3 females and 3 males from each group, were slaughtered at the age of 42 days and their slaughter and carcass characteristics were determined. Serum biochemistry values were determined with blood samples taken from the quails during slaughter. As a result of the study, it was determined that the inhalation method did not affect the slaughter and carcass characteristics of 28–42 day-old Texas quails, but it had a reducing effect on HDL, triglyceride, and glucose values, which indicate lipid and carbohydrate metabolism, among the serum biochemistry values examined. Considering the positive effects of the inhalation of lavender essential oil on animal health, it is recommended to conduct more comprehensive studies.

## Introduction

Quails have earlier development ability, shorter generation intervals, and shorter incubation periods compared to other poultry species. Owing these features, they provide important animal protein sources such as meat and eggs in a short time (Lukanov and Pavlova, 2020; El-Hindawy et al., 2021; Ramankevich et al., 2022; Ismael et al., 2024). Poultry has a model animal; therefore, it also has an important place in experimental studies (Morris et al.., 2020). Texas quail is a meat-type genotype. Although it is heavier than Japanese quail, it reaches slaughter maturity later (Şengül et al., 2021; Volkova et al., 2023). Number of studies on Texas quails is quite limited.

The most important factors contributing to the sustainability of poultry farming are protecting animal health and improving growth performance. For this purpose, new, affordable, and applicable alternative solutions need to be found in the poultry industry.

A lot of research on the application of natural bioactive components obtained from plants in poultry has attracted attention (Toghyani et al., 2010; Shirani et al., 2019; Reda et al., 2020; Daş et al., 2020; Behboodi et al., 2021). Essential oils (EOs) extracted from different parts of medicinal aromatic plants contain various volatile and aromatic components (Bakkali et al., 2008; Besharati et al., 2021). In poultry farming, EOs exhibit beneficial effects on the preservation of animal health as well as growth performance, slaughter, and carcass characteristics (Küçükyilmaz et al., 2017; Barbarestani et al., 2020; Daş et al., 2020; Alagawany et al., 2021; Özbilgin et al., 2023). Lavender (*Lavandula angustifolia*) is a perennial and aromatic plant belonging to the Lamiaceae family. This plant has a semi-shrub form, a height of 20 to 60 cm and lilac and blue flowers (Zeybek and Zeybek, 1994; Ceylan, 1997; Özbilgin et al., 2023). Lavender essential oil (LEO) is extracted from the flowers of the plant and has colourless or pale yellow. The quality and market value of LEO depend on its composition. High levels of linalool and linalyl acetate and low levels of camphor are crucial for high quality essential oil (Ayran Çolak and Çelik, 2023). LEO is commonly used in aromatherapy, perfumes, cosmetics, and food products (Ceylan, 1997; Giray, 2018). It has calming effects on the nerves and can help deal with insomnia problems (Sönmez et al., 2018) due to its pleasant and relaxing scent. LEO contains have been widely studied and has a therapeutic effect (Chrysargyris et al., 2016; Xylia et al., 2017).

Due to the rich terpene content of this plant, it has anti-inflammatory, antiviral, antioxidant, and antibacterial properties and may have positive effects on the growth and development of animals (Küçükyilmaz et al., 2017; Salarmoini et al., 2019; Barbarestani et al., 2020; Adaszyńska-Skwirzyńska et al., 2021; Behboodi et al., 2021).

Lavender essential oil (LEO) contains a high level of monoterpenes and especially linalool and linalyl acetate are the most active volatile components in the oil (Gonçalves and Romano, 2013; Salarmoini et al., 2019). It is known that volatile components bind to odour receptors as a result of inhalation, causing signal transmission in the brain and this odour stimulation can affect the central and autonomic nervous systems. Changes in these systems lead to changes in sympathetic and parasympathetic nerve activities and affect energy and lipid metabolism (Kim et al., 2022; Hong et al., 2024).

It has been previously investigated how addition of LEO into diets or drinking water affects growth performance and lipid metabolism in poultry species (Küçükyilmaz et al., 2017; Salarmoini et al., 2019; Barbarestani et al., 2020; Adaszyńska-Skwirzyńska et al., 2021; Behboodi et al., 2021; Özbilgin and Kara, 2023). However, the use of the inhalation method in poultry breeding to benefit from the health and growth performance-improving effect of LEO has not been studied. This study was designed considering the metabolic effects of inhalation of volatile active compounds in plant extracts.

The aim of this study is to investigate the effect of inhalation of LEO on slaughter and carcass characteristics and serum biochemistry values cholesterol, high-density lipoproteins cholesterol (HDL), low density lipoproteins cholesterol (LDL), very low-density lipoprotein, cholesterol (VLDL), triglycerides, and glucose (GLU) values in Texas quails.

## Material and method

### Ethics Considerations

The animal study protocol was approved by Selçuk University Experimental Research and Application Centre, Animal Experiments Ethics Committee (Ethics Committee Decision Number: 2024/131) for studies involving animals. Working Order, Experimental Design, Maintenance, and Feeding This study was carried out at Selçuk University Prof. Dr. Hümeyra ÖZGEN Research and Application Farm. As a result of the Power test, 36 Texas quails were determined to be used as materials for each group.

In this study, 72 Texas quails purchased from a private enterprise at the age of 1 day constituted the material of the study. The quails were raised together under the same care and feeding conditions until 28 days of age. As of the fourth week of age, the animals were divided into two groups: the control and lavender groups. The arrangement of this study is two experiments and two controls, and also 18 animals in each subgroup were raised in breeding cages of groups until 42 days of age (Table 1).

**Table 1:** Component of quail diet.

Component	Value
Crude protein (%)	20
Crude cellulose (%)	4.5
Crude ash (%)	6.80
Crude fat (%)	4.80
Lysine (%)	1.40
Methionine (%)	0.50
Vit A (IU/Kg)	13440
Vit D3 (IU/Kg)	3200
Vit E (IU/Kg)	79.6
Ca (%)	0.70
P (%)	0.60
Na (%)	0.20

The average live weight of the quails at the 4th week was 148.36 g. The quails were randomly selected for each group according to the zigzag method (Inal, 2005). Lavender oil inhalation prepared at 0.1 % was applied to the lavender group for 1 h a day for 14 days. The quails were housed in 4×4 m rearing rooms in the alternative poultry unit. During their care, the animals were subjected to a 23-hour light and 1-hour dark lighting cycle. All the quails were given access to water and feed ad libitium (Table 1) when the animals were 42 days old, 3 female and 3 male animals from each group were randomly selected and slaughtered. Blood biochemistry values (Alagavany et al., 2021) and slaughter and carcass characteristics of the animals selected for slaughter were identified.

### Cultivation of Lavender

Lavender is a plant that is widely distributed in Mediterranean and belongs to the Lamiaceae family. This plant is not found naturally in the geography of Türkiye and has narrow leaves, semi-shrub form, and lilac flowers. Lavender is a plant that can be produced vegetatively and generatively (seeds). Due to foreign pollination of the plant, the use of cuttings and rooted shoots as vegetative parts in its cultivation are common. In this study, the ‘Hemus’ lavender variety, which belongs to the *Lavandula angustifolia* species and is currently cultivated was used. The quality of lavender production is largely determined by harvest and post-harvest processes. The flowers must be dried in a cool and well-ventilated area at a temperature that will not cause any loss of colour or essential oils. Lavender flowers are harvested during the full flowering period, typically in June and July. In July 2023, the lavender plants in the study were manually harvested during the full flowering period. The harvested flowers were then dried under suitable conditions and stored for the extraction of LEO.

### Distillation and GC-MS Analysis of Essential Oils

Dried and powdered lavender flowers were weighed as 100 grams and hydro distilled with 500 ml of water for 3.5 h using the Clevenger apparatus. Content analyses of the essential oils were measured using a GC-MS device. Conditions of the analysis procedure to determine the components of LEO: Column: DB-WAX 60 x 0.25 x 0.25; Flow: 1.5 ml/min He; Inlet temperature: 250°C; Split ratio: 40:1; Injection volume: 1 μL; MSD transfer line temperature: 250°C; MS welding temperature: 230°C; MS quadrupole temperature: 150°C; FID temperature: 220°C; FID dry air: 400 ml/min; and FID hydrogen: 30 ml/minute (Ayran Colak and Celik, 2023).

### Measurement of Biochemical Parameters

Blood samples were collected from six Texas quails selected from each group (lavender/control) on the 42nd day of age. Samples were centrifuged at 3.500 rpm for 15 min for serum separation and were stored at –20 °C until they were analysed (Alagawany et al., 2021).

The serum concentration of cholesterol, high-density lipoproteins cholesterol (HDL), low-density lipoproteins cholesterol (LDL), very low-density lipoprotein cholesterol (VLDL), triglycerides, and glucose values were determined by spectrophotometric methods in accordance the instructions of the kit's manufacturer (bt PRODUCT, İzmir, Türkiye).

### Determination of Slaughter and Carcass Characteristics

Six quails from each of the lavender and control groups were randomly selected, regardless of sex, and their live weight values were determined using a digital scale with a sensitivity of 0.01 g before slaughter. Animals selected from both groups were cut with a knife from *V.jugularis* to examine their slaughter and carcass characteristics, letting the blood drain for 5 min. After the feathers, head, feet, and internal organs of the quails were removed according to the appropriate slaughtering method, their carcass weight as well as their heart weight, lung weight, gizzard weight, and liver weight values from the slaughter characteristics were determined with a precision scale and numbered.

The following formula was used to calculate carcass yield

Carcass yield (%) = (Carcass weight/Live weight before slaughter) *100

### Statistical analysis

Power analysis was performed using G*Power (version 3.1.9.7) programme and type 1 error probability (alpha) = 0.05, power (1-beta) = 0.80, effect size (dz = 0.50) (Cohen, 1988), H0: µ = µ0, H1: µµ0 criteria to calculate the required minimum sample size before the study. The required minimum sample size was calculated to be 64.

In this study, the measurements obtained in mg/dl were first converted to mmol/L to make statistical evaluations. Whether or not all data are normally distributed was checked with the Shapiro-Wilk test, and the homogeneity of variances was checked with Levene's test. As a result of the evaluations, the independent samples t-test and Mann-Whitney U test were used to compare two independent groups depending on whether the data met the parametric test assumptions or not. Data were presented as n, *Mean±SEM*, and p-value. Statistical analysis was performed using SPSS software (Version 29).

## Results

Table 2 shows chemical composition of LEO.

**Table 2:** Chemical composition of essential oil of lavender (%).

Compound	RI	Amount (%)
Amylethyl ketone	984	1,65
Myrcene	989	0,72
Butanoic acid	994	0,74
Acetic acid	1010	1,16
Limonene	1030	0,5
1,8 cineole	1033	1,35
cis-Ocimene	1035	1,17
β-Ocimene	1046	1,21
Linalool oxide	1073	0,73
**Linalool**	**1108**	**40,67**
Lavandulol	1167	1,04
Endo-borneol	1179	0,92
3-cyclohexen-1-ol	1187	3,14
Cryptone	1192	1,02
α-Terpineol	1202	3,54
Nerol	1229	0,61
**Linalyl acetate**	**1253**	**26,22**
Lavandulyl acetate	1283	5,3
Neryl acetate	1357	1,07
Geranyl acetate	1377	1,58
β-caryophyllene	1429	0,76
Farnesene	1453	0,78
Caryophyllene oxide	1595	0,83

The essential oil yield of *Lavandula angustifolia* was measured as 3.6 %. Essential oil components of 0.5 % and greater are given in Table 2, and the total of the detected essential oil components was 100 %. According to the International Organization for Standardization (ISO 3515:2002), the composition of LEO had a linalool rate of 25.0-38.0 %, a linalyl acetate rate of 25.0-45.0 %, a cymene rate of 4.0-10.0 %, a 4-terpineol rate of 2.0-6.0 % and a camphor rate of 0-0.5 % (Anonymous, 2002). Especially European Pharmacopoeia (2011) states that the camphor rate is 1.2 %.

Table 3 shows biochemical measurements of 42 day-old Texas quails.

**Table 3:** Biochemical measurements of 42 day-old Texas quail.

Variable	Group	N	Mean±SEM	p-value
VLDL	C	6	0.93±0.30	0.485
	L	6	0.78±0.10	
HDL	C	6	3.33±0.50	0.022
	L	6	6.01±0.86	
LDL	C	6	1.52±0.26	0.160
	L	6	2.35±0.49	
CHOLESTEROL	C	6	7.75±0.60	0.080
	L	6	8.97±0.20	
TRIGLYCERIDE	C	6	5.08±1.38	0.009
	L	6	2.70±0.14	
GLUCOSE	C	6	19.44±0.46	0.026
	L	6	17.89±0.36	

Measurements of statistically significant biochemical parameters in the control and lavender groups were represented with box plots. As a result of using lavender essential oil by inhalation in poultry houses, a significant increase was observed in HDL and this increase was statistically significant. Triglyceride and glucose values statistically significantly decreased (*p* > 0.05). A statistically significant difference was found in HDL, triglyceride, and glucose parameters between the control and lavender groups (*p* < 0.05) (Table 3).

**Figure 1:**
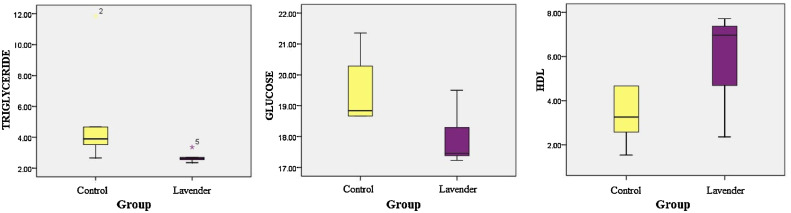
Box plot graphs of biochemical parameters.

Fig. 1 shows box plot graphs of biochemical parameters.

As a result of the statistical evaluation of body weight measurements of Texas quails, no statistical difference was found between the control and lavender groups (*p* > 0.05) (Table 4).

**Table 4:** Slaughter and carcass characteristics of Texas quails.

Variable	Group	n	Mean±SEM	p-value
LWBS (g)	C	6	267.50± 6.29	0.672
	L	6	263.60 ± 6.36	
Carcass weight (g)	C	6	184.13±3.08	0.601
	L	6	181.56±3.66	
Carcass yield (%)	C	6	68.93±1.08	1.000
	L	6	68.92±0.70	
Heart weight (g)	C	6	2.42±0.05	0.461
	L	6	2.58±0.21	
Lung weight (g)	C	6	2.42±0.14	0.271
	L	6	2.07±0.27	
Gizzard weight (g)	C	6	4.74±0.30	0.929
	L	6	4.70±0.26	
Liver weight (g)	C	6	5.98±0.22	0.623
	L	6	5.82±0.24	

C: Control, L: LEO, LWBS: Live weight before slaughter.

## Discussion

Lavender essential oil is characterized by a complex chemical composition that contributes to its distinctive aroma and therapeutic effects. The main components of LEO include linalool and linalyl acetate. It was determined that the linalool rate was 40.67 % and the linalyl acetate rate was 26.27 %. The camphor rate (0.28 %) was less than 0.5 %. The components that determine the quality of lavender essential oil namely linalool, linalyl acetate, and camphor (Hui et al., 2010) was determined within the desired ranges in the study.

Cymene and 4-terpineol components were detected as less than 0.5 %. Linalool is known for its antimicrobial and calming properties, while linalyl acetate significantly contributes to the unique fragrance of lavender oil and exhibits stress-relieving effects (Kim and Lee, 2020; Huang et al., 2021). Additionally, lavandulyl acetate enhances the olfactory profile of the oil. Together, these three compounds account for the majority of lavender oil's characteristic scent and therapeutic qualities (Mahmoudi et al., 2022).

Adaszyńska-Skwirzyńska and Szczerbińska (2019), who examined the effects of LEO added to drinking water in broiler chickens, found that the live weights measured in the LEO applied groups between days 1-42 and days 22-42 were higher compared to the control group (*p* < 0.01). They also reported that LEO supplementation had a positive effect on live weight in the second period of rearing (days 22-24). Barberastani et al. (2020) reported that LEO supplementation in diet did not affect the live weight gain in 0-21-day-old broiler chickens.

Researchers reported that the live weight of broiler chickens fed with 600 mg/kg LEO and virginiamycin (VIM) at the ages of 21-42 days was higher than the live weight of 300 mg/kg LEO supplement and control groups (*p* < 0.05). The lack of a difference between groups in this study may be due to species differences, application duration, and application type.

Özbilgin et al. (2023), who examined the effects of adding different doses of LEO to the diet on the live weight of Japanese quails, reported that it had no effect on live weight on the 35th day. In this study, no statistical difference was detected in terms of pre-slaughter live weight values in Japanese quails administered LEO inhalation at the ages of 28-42 days. Laghouati et al. (2020) reported that the addition of LEO to the ration at 0-42 days of age did not affect live weight, but increased carcass weight and carcass ratio compared to the control group. In the same study, the researchers stated that liver weight decreased with the administration. The lack of an effect on slaughter and carcass characteristics in this study conducted on Texas quails may be due to genotype or application differences.

LEO has some important volatile components (monoterpenes etc.). In this study, an increase in HDL levels and a decrease in TG and GLU levels were observed in quail groups exposed to lavender oil essential inhalation (*p* < 0.05). No significant change was observed in VLDL, LDL, and cholesterol levels in the lavender compared to the control group (*p* > 0.05). Some studies have reported that LEO affects blood components (Baberestani et al., 2020; Adaszyńska-Skwirzyńska et al., 2021; Behboodi et al., 2021). In their study, Behboodi et al. (2021) added a new mixture of different substances, including lavender, to the drinking water of broiler chickens under heat stress at certain levels and observed that TG and cholesterol levels lowered in the high-concentration treatment groups compared to the control groups (*p* > 0.05). In all application groups, VLDL and LDL concentrations were lower and HDL concentrations were higher compared to the control group (*p* < 0.05). These findings proved that the relevant extracts and essential oils changed the course of metabolism by affecting blood components. However, another study reported that LEO added to the drinking water of broiler chickens did not create a statistically significant change on the biochemical indexes of the blood (cholesterol, GLU, and TG) (Adaszyńska-Skwirzyńska et al., 2021). Likewise, Mokthari et al. (2018) showed that LEO (100-800 mg/kg) applied as a feed additive did not show any difference in TG, cholesterol, HDL, LDL, TP, and uric acid parameters compared to the control groups, but only a decrease in GLU concentration. It is clear here that the dosage of the essential oil and/or the type of nutrient to which it is added or the method of administration (oral/inhalation) are determining factors in the level of the relevant components.

**Figure 2:**
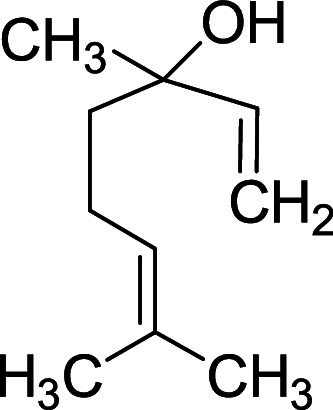
Linalool structure.

Organic compounds obtained from aromatic plants are essentially terpene in their structure, and these terpene structures are responsible for the important bioactive properties attributed to essential oils (Preira et al., 2018). Linalool (C10H18O), one of the main components of lavender, is a type of monoterpene alcohol. Fig. 2 shows its structure. The hydroxyl group in the chemical structure of linalool gives polarity to the compound and the functional groups and double bonds in the linalool structure make it prone to chemical modifications such as oxidation, glycosylation, and methylation (Preira et al., 2018). With these properties, linalool can easily interact with blood components.

It was observed that Linalool, a lavender oil component administered orally to mice for 6 weeks, significantly reduced total cholesterol, LDL, and TG levels compared to control group. Linalool administered at both low and high concentrations elevated HDL levels, and based on all these findings, the relevant compound exhibited hypocholesterolemic activity. The hypocholesterolemic effect of linalool takes mainly place through decreased expression of 3- hydroxy‑3-methyl glutaryl-CoA reductase (HMGCR), which is a marker of hepatic cholesterol synthesis (Cho et al., 2011). De novo cholesterol biosynthesis occurs as a result of the active operation of the mevalonate pathway (MVA), which includes many enzymatic reactions. Conversion of 3-β‑hydroxy-3β-methyl glutaryl CoA (HMG-CoA) to MVA by HMGCR is a key and rate-limiting important step of this synthesis (Martella et al., 2023). In their study Martella et al., reported that LEO had a modulatory effect on cholesterol levels and reduced the expression of HMGCR, an important enzyme involved in cholesterol synthesis and this effect may be mediated by the induction of the negative feedback mechanism induced by intracellular cholesterol accumulation. In another study, 300 and 600 mg/kg lavender oil was administered orally to broiler chickens, and serum cholesterol and LDL-C concentrations decreased (*p* < 0.05) compared to the control and the 600 mg/kg group. On the other hand, serum TG, VLDL-C, HDL-C, TP, and GLU levels were not affected. The reason for the low cholesterol level in animals fed a diet supplemented with high-concentration lavender oil is attributed to the increase in lactic acid bacteria in the intestine and the inhibition of HMG- CoA reductase activity caused by this increase (Baberestani et al., 2020). Linalool suppresses novo lipogenesis and supports fatty acid oxidation, reducing lipid accumulation in the liver in rats fed a high-fat diet (Tamilmani et al., 2023). Even at low concentrations, the relevant monoterpene can block the steps involved in cholesterol synthesis in some cells. At high concentrations, it causes strong inhibition of cholesterogenesis by downregulating HMGCR levels (Kladniew et al., 2014).

In this study, the significant decrease in HDL levels in the lavender group compared to the control group is compatible with the literature. The volatile terpene components contained in LEO may have exhibited hypocholesterolemic activity by acting on MVA. The decrease in TG levels in the same group indicates the modulatory effect of the components contained in essential oils on lipid metabolism, based on the literature. The significant decrease in GLU levels in the groups in the present study indicates that volatile components can regulate energy metabolism via inhalation. Although there was no significant difference in LDL and cholesterol levels, it is thought that a longer inhalation application will change the levels of the relevant parameters more clearly. In addition, examining the changes in the transcription level and protein variations of the relevant enzymes and molecules that play a key role in cholesterogenesis and blood parameters, will provide a more complementary profile for future studies.

## Conclusions

Contrary to studies reporting the positive effects of adding LEO to rations, this study found that the LEO inhalation did not statistically affect slaughter and carcass characteristics. In addition, LEO inhalation applied to Texas quails raised for meat production exhibited significant bioactivities through the relevant components in its content. As a result, it was found that although LEO applied by the inhalation method did not significantly affect meat yield, this method can be used in the treating hypercholesterolemia and controlling live weight in terms of poultry health.

## Declaration of competing interest

None

## Appendix Supplementary materials

Image, application 1
