# Supplementary material for: Effect of inhalation application of lavender essential oil on slaughter and carcass characteristics and serum biochemistry in Texas quail
Source: Poult Sci. 2025 Mar 29;104(6):104828. doi: 10.1016/j.psj.2025.104828 (PMC12018182; doi:10.1016/j.psj.2025.104828)
Supplement: Supplementary file 1 [file mmc1.pdf]

YAKAMOZ çeviri

YAKAMOZ TRANSLATION SERVICES

Certificate of Proofreading

This document certifies that the manuscript listed below was proofread for proper grammar, spelling, punctuation, and overall style by Emel Zindan De Camillis, one of *Yakamoz Translation Bureau's* English-speaking editors/proof-readers:

Manuscript Title: [Effect of Inhalation Application of Lavender Essential Oil on Slaughter and Carcass Characteristics and Serum Biochemistry in Texas Quail**]**

Author(s): [Emre ARSLAN, İrem AYRAN ÇOLAK, İrem BAYAR, Rahile ÖZTÜRK, Sadiye Ayşe ÇELİK, Tuba BAYİR**]**

Date: October 22, 2024

YAKAMOZ TRANSLATION BUREAU

YAKAMOZ TERCÜME OFİSİ
Emel Zindan De Camillis
1683 Sk. No:47/13 Karşıyaka - İZMİR
Karşıyaka V.D. T.C. 100 829 657 08
Tel: 0232 464 79 65

Address: Alaybey Mh. 1683 Sk. No:47 D:13 Karşıyaka– İZMİR
Phone: 0232 464 79 65 Web: www.yakamozceviri.net Karşıyaka T.O. 10082965708
